# Abstract Withdrawn

**DOI:** 10.1186/1757-1146-5-S1-K4

**Published:** 2012-04-10

## Abstract Withdrawn

